# Supplementary material for: Privacy risks of whole-slide image sharing in digital pathology
Source: Nat Commun. 2023 May 4;14:2577. doi: 10.1038/s41467-023-37991-y (PMC10160114; doi:10.1038/s41467-023-37991-y)
Supplement: Supplementary file 3 — Source Data [file 41467_2023_37991_MOESM3_ESM.zip › graphs.nb.html]

WSI Anonymity Statistics


Code 

- Show All Code
- Hide All Code
- Download Rmd

# WSI Anonymity Statistics

Initialization:


Jittered box plots for scanners (not used in the paper) and consecutive slides (Figure 4 - consecutive-boxplot.pdf):


```
aspectr = 0.5
boxwidth = .3
dataScannersEnhanced  %>% ggplot(aes(x=result, y=extractor, fill=extractor)) + stat_summary(fun.data=f, geom="boxplot",width=boxwidth) + stat_boxplot(geom='errorbar',coef=10,width=boxwidth) + stat_summary(fun.data = median_cl_boot, geom = "errorbar", colour = "black", linetype="21", width=.7) + geom_point(pch=21,position = position_jitter(width=0, height = .4),alpha=jitterpointalpha) + labs(x=expression(R[s]), y="Extractor") + coord_cartesian(xlim = c(0.0,1.0), clip="off") + theme(aspect.ratio=aspectr, legend.position = "none")
```


```
[1] "All values of t are equal to  1 \n Cannot calculate confidence intervals"
[1] "All values of t are equal to  1 \n Cannot calculate confidence intervals"
```


```
ggsave("scanners-boxplot.pdf", device=cairo_pdf, width=pagewidth, height=aspectr*pagewidth, units=pagewidthunit, scale=1.3, dpi=600)
```


```
[1] "All values of t are equal to  1 \n Cannot calculate confidence intervals"
[1] "All values of t are equal to  1 \n Cannot calculate confidence intervals"
```


```
dataConsecutiveEnhanced %>% ggplot(aes(x=result, y=extractor, fill=extractor)) + stat_summary(fun.data=f, geom="boxplot",width=boxwidth) + stat_boxplot(geom='errorbar',coef=10,width=boxwidth) + stat_summary(fun.data = median_cl_boot, geom = "errorbar", colour = "black", linetype="21", width=.7) + geom_point(pch=21,position = position_jitter(width=0, height = .4),alpha=jitterpointalpha) + labs(x=expression(R[s]), y="Extractor") + coord_cartesian(xlim = c(0.65,1.0), clip="off") + theme(aspect.ratio=0.5, legend.position = "none")
ggsave("consecutive-boxplot.pdf", device=cairo_pdf, width=pagewidth, height=aspectr*pagewidth, units=pagewidthunit, scale=1.3, dpi=600)
```


```
[1] "All values of t are equal to  0.980132450331126 \n Cannot calculate confidence intervals"
[1] "All values of t are equal to  0.993377483443709 \n Cannot calculate confidence intervals"
```


For scanners we are eventually presenting number of missed matches directly (Figure 3 - scanners-barplot.pdf).


```
aspectr = .8
dataScannersEnhanced %>% ggplot(aes(x=result)) + geom_bar(width=.02,aes(fill=extractor)) + geom_text(stat='count', aes(y=..count..,label=..count..), vjust=-.5, size=2.5) + geom_text(aes(y=-3,label=misses), vjust=1, size=3.0) + coord_cartesian(xlim = c(0.6,1.05), clip="off") + scale_x_continuous(breaks = seq(0.1, by=0.1)) + scale_y_continuous(expand = expansion(mult = c(.1, .2))) + guides(fill = guide_legend(reverse = TRUE)) + facet_wrap(~fct_rev(extractor), ncol=3, drop=FALSE) + labs(x=expression(R[s]), y="Result count") + theme(aspect.ratio=aspectr)
ggsave("scanners-barplot.pdf", device=cairo_pdf,  width=pagewidth, height=.7*aspectr*pagewidth, units=pagewidthunit, scale=1.3, dpi=600)
```


Visualization of distance measures - a boxplot for full WSIs (Figure 5 - distances-boxplot.pdf) and a boxplot for cropped WSIs (Figure 10 - distances-crop-boxplot.pdf). Note that violin plots are not used in the paper as they are less common type of visualization and require higher resolution to be viewed properly.


```
boxwidth = .5

aspectr = 0.7
dodge = 0.85
dataDistances %>% mutate(group = paste(distance,extractor,sep='-')) %>% group_by(distance) %>% ggplot(aes(x=factor(distance), y=result,fill=extractor)) + stat_summary(fun.data=f, geom="boxplot",position = position_dodge(width=dodge),width=boxwidth) + stat_boxplot(geom='errorbar',coef=10,position = position_dodge(width=dodge),width=boxwidth) + stat_summary(fun.data = median_cl_boot, geom = "errorbar", colour = "black", linetype="21",position = position_dodge(width=dodge), width=1.2) + geom_point(pch = 21, position = position_jitterdodge(jitter.width = .1, dodge.width = dodge), alpha=jitterpointalpha) + coord_flip() + scale_x_discrete(labels=c("3" = "3 mm (7.0 p/p) ", "6" = "6 mm (5.3 p/p)", "9" = "9 mm (4.0 p/p)", "12" = "12 mm (2.7 p/p)", "15" = "15 mm (2.1 p/p)", "18" = "18 mm (1.4 p/p)")) + labs(y=expression(R[s]), x=expression("Min. distance threshold "~italic(l)~"(avg number of probes per patient)")) + guides(fill = guide_legend(reverse = TRUE, direction = "vertical")) + theme(aspect.ratio=aspectr)
ggsave("distances-boxplot.pdf", device=cairo_pdf, width=pagewidth, height=aspectr*pagewidth, units=pagewidthunit, scale=1.5, dpi=600)
```


```
aspectr = 3.0
dodge = 0.9
dataDistances %>% mutate(group = paste(distance,extractor,sep='-')) %>% group_by(distance) %>% ggplot(aes(x=factor(distance), y=result,fill=extractor)) + geom_violin(position = position_dodge(width = dodge)) + geom_boxplot(outlier.shape = NA,position = position_dodge(width = dodge),alpha=.4) + stat_summary(fun.data = median_cl_boot, geom = "errorbar", colour = "black", linetype="21",position = position_dodge(width=dodge), width=1.2)  + geom_point(pch = 21, position = position_jitterdodge(jitter.width = .1, dodge.width = dodge), alpha=jitterpointalpha) + coord_flip() + scale_x_discrete(labels=c("3" = "3 mm (7.0 p/p) ", "6" = "6 mm (5.3 p/p)", "9" = "9 mm (4.0 p/p)", "12" = "12 mm (2.7 p/p)", "15" = "15 mm (2.1 p/p)", "18" = "18 mm (1.4 p/p)")) + labs(y=expression(R[s]), x=expression("Min. distance threshold "~italic(l)~"(avg number of probes per patient)")) + guides(fill = guide_legend(reverse = TRUE)) + theme(aspect.ratio=aspectr)
ggsave("distances-violinplot.pdf", device=cairo_pdf, width=pagewidth, height=aspectr*pagewidth, units=pagewidthunit, scale=2.0, dpi=600)
```


```
aspectr = 0.7
dodge = 0.85
dataCropDistances %>% mutate(group = paste(distance,extractor,sep='-')) %>% group_by(distance) %>% ggplot(aes(x=factor(distance), y=result,fill=extractor)) + stat_summary(fun.data=f, geom="boxplot",position = position_dodge(width=dodge),width=boxwidth) + stat_boxplot(geom='errorbar',coef=10,position = position_dodge(width=dodge),width=boxwidth) + stat_summary(fun.data = median_cl_boot, geom = "errorbar", colour = "black", linetype="21",position = position_dodge(width=dodge), width=1.2) + geom_point(pch = 21, position = position_jitterdodge(jitter.width = .1, dodge.width = dodge), alpha=jitterpointalpha) + coord_flip() + scale_x_discrete(labels=c("3" = "3 mm (7.0 p/p) ", "6" = "6 mm (5.3 p/p)", "9" = "9 mm (4.0 p/p)", "12" = "12 mm (2.7 p/p)", "15" = "15 mm (2.1 p/p)", "18" = "18 mm (1.4 p/p)")) + labs(y=expression(R[s]), x=expression("Min. distance threshold "~italic(l)~"(avg number of probes per patient)")) + guides(fill = guide_legend(reverse = TRUE, direction = "vertical")) + theme(aspect.ratio=aspectr)
ggsave("distances-crop-boxplot.pdf", device=cairo_pdf, width=pagewidth, height=aspectr*pagewidth, units=pagewidthunit, scale=1.5, dpi=600)
```


```
aspectr = 3.0
dodge = 0.9
dataCropDistances %>% mutate(group = paste(distance,extractor,sep='-')) %>% group_by(distance) %>% ggplot(aes(x=factor(distance), y=result,fill=extractor)) + geom_violin(position = position_dodge(width = dodge)) + geom_boxplot(outlier.shape = NA,position = position_dodge(width = dodge),alpha=.4) + stat_summary(fun.data = median_cl_boot, geom = "errorbar", colour = "black", linetype="21",position = position_dodge(width=dodge), width=1.2) + geom_point(pch = 21, position = position_jitterdodge(jitter.width = .1, dodge.width = dodge), alpha=jitterpointalpha) + coord_flip() + scale_x_discrete(labels=c("3" = "3 mm (7.0 p/p) ", "6" = "6 mm (5.3 p/p)", "9" = "9 mm (4.0 p/p)", "12" = "12 mm (2.7 p/p)", "15" = "15 mm (2.1 p/p)", "18" = "18 mm (1.4 p/p)")) + labs(y=expression(R[s]), x=expression("Min. distance threshold "~italic(l)~"(avg number of probes per patient)")) + guides(fill = guide_legend(reverse = TRUE)) + theme(aspect.ratio=aspectr)
ggsave("distances-crop-violinplot.pdf", device=cairo_pdf, width=pagewidth, height=aspectr*pagewidth, units=pagewidthunit, scale=2.0, dpi=600)
```


Calculating success rate stats for non-consecutive slides (= max in the table). Note that these tables are not directly used in the paper.


```
dataNumberPatients %>% filter(extractor=="SimCLRv2") %>% group_by(number_of_patients) %>% summarize_at(vars(result), list(min=min, Q1=~quantile(., probs = 0.25), median=median, Q3=~quantile(., probs = 0.75), max=max))
```


```
dataDistances %>% group_by(distance) %>% summarize_at(vars(result), list(min=min, Q1=~quantile(., probs = 0.25), median=median, Q3=~quantile(., probs = 0.75), max=max))
```


```
dataDistancesEpsilons <- dataDistances %>% group_by(distance) %>% summarize_at(vars(result), list(max=max)) %>% mutate(distance = factor(distance), max = sprintf("%0.3f", round.off(max,3))) %>% rename_at(vars(c("distance", "max")), ~ c("distance [mm]", '$\\varepsilon$'))%>% t()
latexTable <- xtable(dataDistancesEpsilons, caption = "Values of $\\varepsilon$ for non-consecutive slides.", label="tab:results:non-consecutive:epsilon")
#align(latexTable) %<>% str_replace("l", "S[table-format=0.3]|")
#align(latexTable) <- xalign(latexTable)
#digits(latexTable) <- xdigits(latexTable)
#display(latexTable) <- xdisplay(latexTable)
print(latexTable,file="tab-results-nonconsecutive-epsilon.tex",append=T,table.placement = "hbtp",caption.placement="bottom", hline.after=seq(from=0,to=nrow(latexTable),by=1), include.colnames = FALSE, sanitize.text.function = function(x){x})

dataCropDistances %>% group_by(distance) %>% summarize_at(vars(result), list(min=min, Q1=~quantile(., probs = 0.25), median=median, Q3=~quantile(., probs = 0.75), max=max))
```


```
dataCropDistancesEpsilons <- dataCropDistances %>% group_by(distance) %>% summarize_at(vars(result), list(max=max)) %>% mutate(distance = factor(distance), max = sprintf("%0.3f", round.off(max,3))) %>% rename_at(vars(c("distance", "max")), ~ c("distance [mm]", '$\\varepsilon$'))%>% t()
latexTable <- xtable(dataCropDistancesEpsilons, caption = "Values of $\\varepsilon$ for non-consecutive cropped slides.", label="tab:results:non-consecutive:epsilon")
#align(latexTable) %<>% str_replace("l", "S[table-format=0.3]|")
#align(latexTable) <- xalign(latexTable)
#digits(latexTable) <- xdigits(latexTable)
#display(latexTable) <- xdisplay(latexTable)
print(latexTable,file="tab-results-nonconsecutive-crops-epsilon.tex",append=T,table.placement = "hbtp",caption.placement="bottom", hline.after=seq(from=0,to=nrow(latexTable),by=1), include.colnames = FALSE, sanitize.text.function = function(x){x})
```


Visualization of crop shifts (Figure 9 - cropshifts-boxplot.pdf). Note that violin plot is not used in the paper as they are less common type of visualization.


```
aspectr = 0.7
dodge = 0.75
boxwidth = .5
dataCropShifts %>% group_by(shift,extractor) %>% summarize_at(vars(result), list(min=min, Q1=~quantile(., probs = 0.25), median=median, Q3=~quantile(., probs = 0.75), max=max))
```


```
#dataCropShifts %>% mutate(group = paste(shift,extractor,sep='-')) %>% group_by(shift) %>% ggplot(aes(x=factor(shift), y=result,fill=extractor)) + geom_boxplot(outlier.shape = NA,position = position_dodge2()) + geom_point(pch = 21, position = position_jitterdodge(jitter.width = .1), alpha=jitterpointalpha) + coord_flip() + scale_x_discrete(labels=c("0" = "0 px (100%) ", "5" = "5 px (97-98%)", "10" = "10 px (94-96%)", "15" = "15 px (91-93%)", "25" = "25 px (85-89%)", "50" = "50 px (71-78%)", "75" = "75 px (58-67%)", "100" = "100 px (47-55%)")) + labs(y=expression(R[s]), x="Shift (overlap)") + guides(fill = guide_legend(reverse = TRUE)) + theme(aspect.ratio=aspectr)
dataCropShifts %>% mutate(group = paste(shift,extractor,sep='-')) %>% group_by(shift) %>% ggplot(aes(x=factor(shift), y=result,fill=extractor)) + stat_summary(fun.data=f, geom="boxplot",position = position_dodge(width=dodge),width=boxwidth) + stat_boxplot(geom='errorbar',coef=10,position = position_dodge(width=dodge),width=boxwidth)  + stat_summary(fun.data = median_cl_boot, geom = "errorbar", colour = "black", linetype="21",position = position_dodge(width=dodge), width=1.2)  + geom_point(pch = 21, position = position_jitterdodge(jitter.width = .1), alpha=jitterpointalpha*.5) + coord_flip() + scale_x_discrete(labels=c("0" = "0 px (100%) ", "5" = "5 px (97-98%)", "10" = "10 px (94-96%)", "15" = "15 px (91-93%)", "25" = "25 px (85-89%)", "50" = "50 px (71-78%)", "75" = "75 px (58-67%)", "100" = "100 px (47-55%)")) + labs(y=expression(R[s]), x="Shift (overlap)") + guides(fill = guide_legend(reverse = TRUE)) + theme(aspect.ratio=aspectr)
ggsave("cropshifts-boxplot.pdf", device=cairo_pdf, width=pagewidth, height=aspectr*pagewidth, units=pagewidthunit, scale=2.0, dpi=600)
```


```
aspectr = 3.0
dodge = 0.9
dataCropShifts %>% mutate(group = paste(shift,extractor,sep='-')) %>% group_by(shift) %>% ggplot(aes(x=factor(shift), y=result,fill=extractor)) + geom_violin(position = position_dodge(width = dodge))  + geom_point(pch = 21, position = position_jitterdodge(jitter.width = .1), alpha=jitterpointalpha*.5) + coord_flip() + scale_x_discrete(labels=c("0" = "0 px (100%) ", "5" = "5 px (97-98%)", "10" = "10 px (94-96%)", "15" = "15 px (91-93%)", "25" = "25 px (85-89%)", "50" = "50 px (71-78%)", "75" = "75 px (58-67%)", "100" = "100 px (47-55%)")) + labs(y=expression(R[s]), x="Shift (overlap)") + guides(fill = guide_legend(reverse = TRUE)) + theme(aspect.ratio=aspectr)
ggsave("cropshifts-violinplot.pdf", device=cairo_pdf, width=pagewidth, height=aspectr*pagewidth, units=pagewidthunit, scale=2.0, dpi=600)
```


Making fanplots of numbers of patients and slides (Figure 8a - numberpatients-intervalplot.pdf and Figure 8b - numberslides-intervalplot.pdf). Note that the boxplot is only used for consistency check and is not used in the paper.


```
aspectr = 0.5
dodge = 0.75
boxwidth = .5
legendxshift = -.5
legendyshift = -2.0
dataNumberSlides %>% group_by(extractor) %>% ggplot(aes(x=factor(number_of_slides), y=result, fill=extractor)) + stat_summary(fun.data=f, geom="boxplot",position = position_dodge(width=dodge),width=boxwidth) + stat_boxplot(geom='errorbar',coef=10,position = position_dodge(width=dodge),width=boxwidth) + stat_summary(fun.data = median_cl_boot, geom = "errorbar", colour = "black", linetype="21",position = position_dodge(width=dodge), width=1.2) + geom_point(pch = 21, position = position_jitterdodge(jitter.width = .1), alpha=jitterpointalpha)+ labs(y=expression(R[s]), x="Number of probes per patient") + guides(fill = guide_legend(reverse = TRUE)) + theme(aspect.ratio=aspectr)
ggsave("numberslides-boxplot.pdf", device=cairo_pdf, width=pagewidth, height=aspectr*pagewidth, units=pagewidthunit, scale=2.0, dpi=600)
```


```
ggNumberSlides <- dataNumberSlides %>% group_by(extractor) %>% ggplot(aes(x=number_of_slides, y=result, group=extractor)) + geom_fan(aes(fill=extractor,alpha=..Interval..), intervals=c(.5,1.0), alpha=.3,show.legend=FALSE) + geom_interval(intervals=c(0,.5,1.0)) + scale_linetype_manual(values=c('solid','dotted','dashed'), labels=c('Median','Q1-Q3', 'min-max')) + facet_wrap(~fct_rev(extractor), ncol=3, drop=FALSE) + labs(y=expression(R[s]), x="Number of probes per patient") + guides(fill = guide_legend(reverse = TRUE)) + theme(aspect.ratio=aspectr) + theme(legend.direction="horizontal", legend.position="bottom")
#ggplotGrob(ggNumberSlides)
#set_last_plot(reposition_legend(ggNumberSlides, 'bottom right', panel = 'panel-3-2'))
#set_last_plot(reposition_legend(ggNumberPatients, 'center', panel = 'panel-2-2', x=legendxshift, y=legendyshift))
ggNumberSlides
ggsave("numberslides-intervalplot.pdf", device=cairo_pdf, width=pagewidth, height=aspectr*pagewidth, units=pagewidthunit, scale=2.0, dpi=600)
```


```
#(ggNumberPatients <- dataNumberPatients %>% group_by(extractor) %>% ggplot(aes(x=number_of_patients, y=result, group=extractor)) + geom_fan(aes(fill=extractor,alpha=..Interval..), intervals=c(.5,1.0), alpha=.3,show.legend=FALSE) + geom_interval(intervals=c(0,.5,1.0)) + scale_linetype_manual(values=c('solid','dotted','dashed'), labels=c('Median','25-75%', 'min-max')) + facet_wrap(~fct_rev(extractor), ncol=3, drop=FALSE) + labs(y=expression(R[s]), x="Number of patients") + guides(fill = guide_legend(reverse = TRUE)) + theme(aspect.ratio=aspectr))
ggNumberPatients <- dataNumberPatients %>% group_by(extractor) %>% ggplot(aes(x=number_of_patients, y=result, group=extractor)) + geom_fan(aes(fill=extractor,alpha=..Interval..), intervals=c(.5,1.0), alpha=.3,show.legend=FALSE) + geom_interval(intervals=c(0,.5,1.0)) + scale_linetype_manual(values=c('solid','dotted','dashed'), labels=c('Median','Q1-Q3', 'min-max')) + facet_wrap(~fct_rev(extractor), ncol=3, drop=FALSE) + labs(y=expression(R[s]), x="Number of patients") + guides(fill = guide_legend(reverse = TRUE)) + theme(aspect.ratio=aspectr) + theme(legend.direction="horizontal", legend.position="bottom")
#ggplotGrob(ggNumberPatients)
#set_last_plot(reposition_legend(ggNumberPatients, 'bottom right', panel = 'panel-3-2', y=-1.2))
#set_last_plot(reposition_legend(ggNumberPatients, 'center', panel = 'panel-2-2', x=legendxshift, y=legendyshift))
ggNumberPatients
ggsave("numberpatients-intervalplot.pdf", device=cairo_pdf, width=pagewidth, height=aspectr*pagewidth, units=pagewidthunit, scale=2.0, dpi=600)
```


LS0tDQp0aXRsZTogIldTSSBBbm9ueW1pdHkgU3RhdGlzdGljcyINCm91dHB1dDogaHRtbF9ub3RlYm9vaw0KLS0tDQoNCkluaXRpYWxpemF0aW9uOg0KDQpgYGB7ciBzZXR1cCwgaW5jbHVkZT1GQUxTRX0NCnJlcXVpcmUodGlkeXZlcnNlKQ0KcmVxdWlyZShtYWdyaXR0cikNCmxpYnJhcnkoeHRhYmxlKQ0KbGlicmFyeShnZ2ZhbikNCmxpYnJhcnkoYm9vdCkNCmxpYnJhcnkobGVtb24pDQpsaWJyYXJ5KGV4dHJhZm9udCkNCiNpbnN0YWxsLnBhY2thZ2VzKCJleHRyYWZvbnRkYiIpDQojbGlicmFyeShyZW1vdGVzKQ0KIyBUaGlzIGlzIG5lZWRlZCB0byBhdm9pZCBObyBGb250TmFtZSBwcm9ibGVtDQojcmVtb3Rlczo6aW5zdGFsbF92ZXJzaW9uKCJSdHRmMnB0MSIsIHZlcnNpb24gPSAiMS4zLjgiKQ0KIyBUaGlzIGlzIG9ubHkgbmVlZGVkIGluaXRpYWxseSBvciB3aGVuIGZvbnRzIGNoYW5nZQ0KI2V4dHJhZm9udDo6Zm9udF9pbXBvcnQoKQ0KI2ZvbnR0YWJsZSgpDQpsb2FkZm9udHMoZGV2aWNlID0gIndpbiIpDQpsb2FkZm9udHMoZGV2aWNlID0gInBvc3RzY3JpcHQiKQ0KbG9hZGZvbnRzKGRldmljZSA9ICJwZGYiKQ0KDQp0aGVtZV9zZXQodGhlbWVfYncoYmFzZV9zaXplPTE0LCBiYXNlX2ZhbWlseSA9ICdMaW51eCBCaW9saW51bSBHJykpDQoNCnBhZ2V3aWR0aD0xNDYNCnBhZ2V3aWR0aHVuaXQ9J21tJw0Kaml0dGVycG9pbnRhbHBoYSA9IDAuMTUNCg0Kcm91bmQub2ZmIDwtIGZ1bmN0aW9uICh4LCBkaWdpdHM9MCkgDQp7DQogIHBvc25lZyA9IHNpZ24oeCkNCiAgeiA9IHRydW5jKGFicyh4KSAqIDEwIF4gKGRpZ2l0cyArIDEpKSAvIDEwDQogIHogPSBmbG9vcih6ICogcG9zbmVnICsgMC41KSAvIDEwIF4gZGlnaXRzDQogIHJldHVybih6KQ0KfQ0KDQpvIDwtIGZ1bmN0aW9uKHgpIHsNCiAgc3Vic2V0KHgsIHggPT0gbWF4KHgpIHwgeCA9PSBtaW4oeCkpDQp9DQoNCmYgPC0gZnVuY3Rpb24oeCkgew0KICByIDwtIHF1YW50aWxlKHgsIHByb2JzID0gYygwLjAwLCAwLjI1LCAwLjUsIDAuNzUsIDEpKQ0KICBuYW1lcyhyKSA8LSBjKCJ5bWluIiwgImxvd2VyIiwgIm1pZGRsZSIsICJ1cHBlciIsICJ5bWF4IikNCiAgcg0KfQ0KDQptZWRpYW5fY2xfYm9vdCA8LSBmdW5jdGlvbih4LCBjb25mID0gMC45NSkgew0KICAgIGxjb25mIDwtICgxIC0gY29uZikvMg0KICAgIHVjb25mIDwtIDEgLSBsY29uZg0KICAgIHJlcXVpcmUoYm9vdCkNCiAgICBibWVkaWFuIDwtIGZ1bmN0aW9uKHgsIGluZCkgbWVkaWFuKHhbaW5kXSkNCiAgICBidCA8LSBib290KHgsIGJtZWRpYW4sIDEwMDApDQogICAgYmIgPC0gYm9vdC5jaShidCwgdHlwZSA9ICJiYXNpYyIpDQogICAgZGF0YS5mcmFtZSh5ID0gbWVkaWFuKHgpLCB5bWluID0gcXVhbnRpbGUoYnQkdCwgbGNvbmYpLCB5bWF4ID0gcXVhbnRpbGUoYnQkdCwgDQogICAgICAgIHVjb25mKSkNCn0NCg0KDQpkYXRhU2Nhbm5lcnMgPSByZWFkLmNzdignRmlndXJlLTNfRS0xLmNzdicpDQpkYXRhU2Nhbm5lcnMgJTw+JSBtdXRhdGVfYXQodmFycyhleHRyYWN0b3IpLCB+IHN0cl9yZXBsYWNlKC4sICJjbHIiLCAiU2ltQ0xSdjIiKSkgJT4lIG11dGF0ZV9hdCh2YXJzKGV4dHJhY3RvciksIH4gc3RyX3JlcGxhY2UoLiwgInJlc25ldCIsICJSZXNOZXQiKSkgJT4lIG11dGF0ZV9hdCh2YXJzKGV4dHJhY3RvciksIH4gc3RyX3JlcGxhY2UoLiwgInZnZzE2IiwgIlZHRzE2IikpICU+JSBtdXRhdGVfYXQodmFycyhleHRyYWN0b3IpICwgfiBzdHJfcmVwbGFjZSguLCAiVkdHMTZwcmUiLCAiVkdHMTZoaXN0byIpKSAlPiUgbXV0YXRlX2F0KHZhcnMoZXh0cmFjdG9yKSwgfiBzdHJfcmVwbGFjZSguLCAiaW1nMnZlYyIsICJJbWcydmVjIikpICU+JSBtdXRhdGVfYXQodmFycyhleHRyYWN0b3IpLCB+IHN0cl9yZXBsYWNlKC4sICJpbmNlcHRpb24iLCAiSW5jZXB0aW9uIikpICU+JSBmaWx0ZXIobWV0cmljID09ICJjb3NpbmUiKSAlPiUgbXV0YXRlX2F0KHZhcnMobWV0cmljKSwgfiBzdHJfcmVwbGFjZSguLCAiY29zaW5lIiwgIkNvc2luZSIpKSAlPiUgbXV0YXRlX2F0KHZhcnMobWV0cmljKSwgfiBzdHJfcmVwbGFjZSguLCAibmV3X2V1Y2xpZCIsICJFdWNsaWRlYW4iKSkNCmRhdGFTY2FubmVyc0VuaGFuY2VkID0gZGF0YVNjYW5uZXJzICU+JSBncm91cF9ieShleHRyYWN0b3IsIG1ldHJpYykgJT4lIG11dGF0ZShncm91cD1wYXN0ZShleHRyYWN0b3IsbWV0cmljLHNlcD0iLSIpKSAlPiUgbXV0YXRlKG1pc3NlcyA9IGZhY3Rvcihyb3VuZCgyOCooMS1yZXN1bHQpLCBkaWdpdHM9MCkpKSAlPiUgbXV0YXRlKHJlc3VsdD1hcy5udW1lcmljKHJvdW5kKHJlc3VsdCwgZGlnaXQ9OCkpKQ0KDQpkYXRhQ29uc2VjdXRpdmUgPSByZWFkLmNzdignRmlndXJlLTRfRS0yYS5jc3YnKQ0KZGF0YUNvbnNlY3V0aXZlICU8PiUgbXV0YXRlX2F0KHZhcnMoZXh0cmFjdG9yKSwgfiBzdHJfcmVwbGFjZSguLCAiY2xyIiwgIlNpbUNMUnYyIikpICU+JSBtdXRhdGVfYXQodmFycyhleHRyYWN0b3IpLCB+IHN0cl9yZXBsYWNlKC4sICJyZXNuZXQiLCAiUmVzTmV0IikpICU+JSBtdXRhdGVfYXQodmFycyhleHRyYWN0b3IpLCB+IHN0cl9yZXBsYWNlKC4sICJ2Z2cxNiIsICJWR0cxNiIpKSAlPiUgbXV0YXRlX2F0KHZhcnMoZXh0cmFjdG9yKSAsIH4gc3RyX3JlcGxhY2UoLiwgIlZHRzE2cHJlIiwgIlZHRzE2aGlzdG8iKSkgJT4lIG11dGF0ZV9hdCh2YXJzKGV4dHJhY3RvciksIH4gc3RyX3JlcGxhY2UoLiwgImltZzJ2ZWMiLCAiSW1nMnZlYyIpKSAlPiUgbXV0YXRlX2F0KHZhcnMoZXh0cmFjdG9yKSwgfiBzdHJfcmVwbGFjZSguLCAiaW5jZXB0aW9uIiwgIkluY2VwdGlvbiIpKSAlPiUgZmlsdGVyKG1ldHJpYyA9PSAiY29zaW5lIikgJT4lIG11dGF0ZV9hdCh2YXJzKG1ldHJpYyksIH4gc3RyX3JlcGxhY2UoLiwgImNvc2luZSIsICJDb3NpbmUiKSkgJT4lIG11dGF0ZV9hdCh2YXJzKG1ldHJpYyksIH4gc3RyX3JlcGxhY2UoLiwgIm5ld19ldWNsaWQiLCAiRXVjbGlkZWFuIikpDQpkYXRhQ29uc2VjdXRpdmVFbmhhbmNlZCA9IGRhdGFDb25zZWN1dGl2ZSAlPiUgZ3JvdXBfYnkoZXh0cmFjdG9yLCBtZXRyaWMpICU+JSBtdXRhdGUoZ3JvdXA9cGFzdGUoZXh0cmFjdG9yLG1ldHJpYyxzZXA9IiwiKSkgJT4lIG11dGF0ZShtaXNzZXMgPSBmYWN0b3Iocm91bmQoMTUxKigxLXJlc3VsdCksIGRpZ2l0cz0wKSkpDQoNCmRhdGFEaXN0YW5jZXMgPSByZWFkLmNzdignRmlndXJlLTVfRS0yYi5jc3YnKQ0KZGF0YURpc3RhbmNlcyAlPD4lIG11dGF0ZV9hdCh2YXJzKGV4dHJhY3RvciksIH4gc3RyX3JlcGxhY2UoLiwgImNsciIsICJTaW1DTFJ2MiIpKSAlPiUgbXV0YXRlX2F0KHZhcnMoZXh0cmFjdG9yKSwgfiBzdHJfcmVwbGFjZSguLCAicmVzbmV0IiwgIlJlc05ldCIpKSAlPiUgbXV0YXRlX2F0KHZhcnMoZXh0cmFjdG9yKSwgfiBzdHJfcmVwbGFjZSguLCAidmdnMTYiLCAiVkdHMTYiKSkgJT4lIG11dGF0ZV9hdCh2YXJzKGV4dHJhY3RvcikgLCB+IHN0cl9yZXBsYWNlKC4sICJWR0cxNnByZSIsICJWR0cxNmhpc3RvIikpICU+JSBtdXRhdGVfYXQodmFycyhleHRyYWN0b3IpLCB+IHN0cl9yZXBsYWNlKC4sICJpbWcydmVjIiwgIkltZzJ2ZWMiKSkgJT4lIG11dGF0ZV9hdCh2YXJzKGV4dHJhY3RvciksIH4gc3RyX3JlcGxhY2UoLiwgImluY2VwdGlvbiIsICJJbmNlcHRpb24iKSkgJT4lIGZpbHRlcihtZXRyaWMgPT0gImNvc2luZSIpICU+JSBtdXRhdGVfYXQodmFycyhtZXRyaWMpLCB+IHN0cl9yZXBsYWNlKC4sICJjb3NpbmUiLCAiQ29zaW5lIikpICU+JSBtdXRhdGVfYXQodmFycyhtZXRyaWMpLCB+IHN0cl9yZXBsYWNlKC4sICJuZXdfZXVjbGlkIiwgIkV1Y2xpZGVhbiIpKQ0KZGF0YURpc3RhbmNlcyAlPD4lIG11dGF0ZShkaXN0YW5jZSA9IGRpc3RhbmNlICsgMSkNCg0KZGF0YUNyb3BEaXN0YW5jZXMgPSByZWFkLmNzdignRmlndXJlLTEwX2NFLTJiLmNzdicpDQpkYXRhQ3JvcERpc3RhbmNlcyAlPD4lIG11dGF0ZV9hdCh2YXJzKGV4dHJhY3RvciksIH4gc3RyX3JlcGxhY2UoLiwgImNsciIsICJTaW1DTFJ2MiIpKSAlPiUgbXV0YXRlX2F0KHZhcnMoZXh0cmFjdG9yKSwgfiBzdHJfcmVwbGFjZSguLCAicmVzbmV0IiwgIlJlc05ldCIpKSAlPiUgbXV0YXRlX2F0KHZhcnMoZXh0cmFjdG9yKSwgfiBzdHJfcmVwbGFjZSguLCAidmdnMTYiLCAiVkdHMTYiKSkgJT4lIG11dGF0ZV9hdCh2YXJzKGV4dHJhY3RvcikgLCB+IHN0cl9yZXBsYWNlKC4sICJWR0cxNnByZSIsICJWR0cxNmhpc3RvIikpICU+JSBtdXRhdGVfYXQodmFycyhleHRyYWN0b3IpLCB+IHN0cl9yZXBsYWNlKC4sICJpbWcydmVjIiwgIkltZzJ2ZWMiKSkgJT4lIG11dGF0ZV9hdCh2YXJzKGV4dHJhY3RvciksIH4gc3RyX3JlcGxhY2UoLiwgImluY2VwdGlvbiIsICJJbmNlcHRpb24iKSkgJT4lIGZpbHRlcihtZXRyaWMgPT0gImNvc2luZSIpICU+JSBtdXRhdGVfYXQodmFycyhtZXRyaWMpLCB+IHN0cl9yZXBsYWNlKC4sICJjb3NpbmUiLCAiQ29zaW5lIikpICU+JSBtdXRhdGVfYXQodmFycyhtZXRyaWMpLCB+IHN0cl9yZXBsYWNlKC4sICJuZXdfZXVjbGlkIiwgIkV1Y2xpZGVhbiIpKQ0KZGF0YUNyb3BEaXN0YW5jZXMgJTw+JSBtdXRhdGUoZGlzdGFuY2UgPSBkaXN0YW5jZSArIDEpDQoNCmRhdGFDcm9wU2hpZnRzID0gcmVhZC5jc3YoJ0ZpZ3VyZS05X2Nyb3Atc2hpZnRzLmNzdicpDQpkYXRhQ3JvcFNoaWZ0cyAlPD4lIG11dGF0ZV9hdCh2YXJzKGV4dHJhY3RvciksIH4gc3RyX3JlcGxhY2UoLiwgImNsciIsICJTaW1DTFJ2MiIpKSAlPiUgbXV0YXRlX2F0KHZhcnMoZXh0cmFjdG9yKSwgfiBzdHJfcmVwbGFjZSguLCAicmVzbmV0IiwgIlJlc05ldCIpKSAlPiUgbXV0YXRlX2F0KHZhcnMoZXh0cmFjdG9yKSwgfiBzdHJfcmVwbGFjZSguLCAidmdnMTYiLCAiVkdHMTYiKSkgJT4lIG11dGF0ZV9hdCh2YXJzKGV4dHJhY3RvcikgLCB+IHN0cl9yZXBsYWNlKC4sICJWR0cxNnByZSIsICJWR0cxNmhpc3RvIikpICU+JSBtdXRhdGVfYXQodmFycyhleHRyYWN0b3IpLCB+IHN0cl9yZXBsYWNlKC4sICJpbWcydmVjIiwgIkltZzJ2ZWMiKSkgJT4lIG11dGF0ZV9hdCh2YXJzKGV4dHJhY3RvciksIH4gc3RyX3JlcGxhY2UoLiwgImluY2VwdGlvbiIsICJJbmNlcHRpb24iKSkgJT4lIGZpbHRlcihtZXRyaWMgPT0gImNvc2luZSIpICU+JSBtdXRhdGVfYXQodmFycyhtZXRyaWMpLCB+IHN0cl9yZXBsYWNlKC4sICJjb3NpbmUiLCAiQ29zaW5lIikpICU+JSBtdXRhdGVfYXQodmFycyhtZXRyaWMpLCB+IHN0cl9yZXBsYWNlKC4sICJuZXdfZXVjbGlkIiwgIkV1Y2xpZGVhbiIpKQ0KDQoNCmRhdGFOdW1iZXJTbGlkZXMgPSByZWFkLmNzdignRmlndXJlLTdiX251bWJlci1zbGlkZXMuY3N2JykNCmRhdGFOdW1iZXJTbGlkZXMgJTw+JSBtdXRhdGVfYXQodmFycyhleHRyYWN0b3IpLCB+IHN0cl9yZXBsYWNlKC4sICJjbHIiLCAiU2ltQ0xSdjIiKSkgJT4lIG11dGF0ZV9hdCh2YXJzKGV4dHJhY3RvciksIH4gc3RyX3JlcGxhY2UoLiwgInJlc25ldCIsICJSZXNOZXQiKSkgJT4lIG11dGF0ZV9hdCh2YXJzKGV4dHJhY3RvciksIH4gc3RyX3JlcGxhY2UoLiwgInZnZzE2IiwgIlZHRzE2IikpICU+JSBtdXRhdGVfYXQodmFycyhleHRyYWN0b3IpICwgfiBzdHJfcmVwbGFjZSguLCAiVkdHMTZwcmUiLCAiVkdHMTZoaXN0byIpKSAlPiUgbXV0YXRlX2F0KHZhcnMoZXh0cmFjdG9yKSwgfiBzdHJfcmVwbGFjZSguLCAiaW1nMnZlYyIsICJJbWcydmVjIikpICU+JSBtdXRhdGVfYXQodmFycyhleHRyYWN0b3IpLCB+IHN0cl9yZXBsYWNlKC4sICJpbmNlcHRpb24iLCAiSW5jZXB0aW9uIikpICU+JSBmaWx0ZXIobWV0cmljID09ICJjb3NpbmUiKSAlPiUgbXV0YXRlX2F0KHZhcnMobWV0cmljKSwgfiBzdHJfcmVwbGFjZSguLCAiY29zaW5lIiwgIkNvc2luZSIpKSAlPiUgbXV0YXRlX2F0KHZhcnMobWV0cmljKSwgfiBzdHJfcmVwbGFjZSguLCAibmV3X2V1Y2xpZCIsICJFdWNsaWRlYW4iKSkNCiMgbnVtYmVyIG9mIHNsaWRlcyBpbiB0aGUgQ1NWIGlzIGFjdHVhbGx5IHRvdGFsIG51bWJlciBwZXIgcGF0aWVudCBhbmQgbm90IG51bWJlciBvZiBwcm9iZXMgLSBvbmUgc2xpZGUgZ29lcyBhbHdheXMgaW50byB0aGUgYmFja2dyb3VuZCBrbm93bGVkZ2UNCmRhdGFOdW1iZXJTbGlkZXMgJTw+JSBtdXRhdGUobnVtYmVyX29mX3NsaWRlcyA9IG51bWJlcl9vZl9zbGlkZXMgLSAxKSAlPiUgZmlsdGVyKG51bWJlcl9vZl9zbGlkZXMgPiAwKQ0KDQpkYXRhTnVtYmVyUGF0aWVudHMgPSByZWFkLmNzdignRmlndXJlLTdhX251bWJlci1wYXRpZW50cy5jc3YnKQ0KZGF0YU51bWJlclBhdGllbnRzICU8PiUgbXV0YXRlX2F0KHZhcnMoZXh0cmFjdG9yKSwgfiBzdHJfcmVwbGFjZSguLCAiY2xyIiwgIlNpbUNMUnYyIikpICU+JSBtdXRhdGVfYXQodmFycyhleHRyYWN0b3IpLCB+IHN0cl9yZXBsYWNlKC4sICJyZXNuZXQiLCAiUmVzTmV0IikpICU+JSBtdXRhdGVfYXQodmFycyhleHRyYWN0b3IpLCB+IHN0cl9yZXBsYWNlKC4sICJ2Z2cxNiIsICJWR0cxNiIpKSAlPiUgbXV0YXRlX2F0KHZhcnMoZXh0cmFjdG9yKSAsIH4gc3RyX3JlcGxhY2UoLiwgIlZHRzE2cHJlIiwgIlZHRzE2aGlzdG8iKSkgJT4lIG11dGF0ZV9hdCh2YXJzKGV4dHJhY3RvciksIH4gc3RyX3JlcGxhY2UoLiwgImltZzJ2ZWMiLCAiSW1nMnZlYyIpKSAlPiUgbXV0YXRlX2F0KHZhcnMoZXh0cmFjdG9yKSwgfiBzdHJfcmVwbGFjZSguLCAiaW5jZXB0aW9uIiwgIkluY2VwdGlvbiIpKSAlPiUgZmlsdGVyKG1ldHJpYyA9PSAiY29zaW5lIikgJT4lIG11dGF0ZV9hdCh2YXJzKG1ldHJpYyksIH4gc3RyX3JlcGxhY2UoLiwgImNvc2luZSIsICJDb3NpbmUiKSkgJT4lIG11dGF0ZV9hdCh2YXJzKG1ldHJpYyksIH4gc3RyX3JlcGxhY2UoLiwgIm5ld19ldWNsaWQiLCAiRXVjbGlkZWFuIikpDQoNCmBgYA0KDQpKaXR0ZXJlZCBib3ggcGxvdHMgZm9yIHNjYW5uZXJzIChub3QgdXNlZCBpbiB0aGUgcGFwZXIpIGFuZCBjb25zZWN1dGl2ZSBzbGlkZXMgKEZpZ3VyZSA0IC0gY29uc2VjdXRpdmUtYm94cGxvdC5wZGYpOg0KDQpgYGB7cn0NCmFzcGVjdHIgPSAwLjUNCmJveHdpZHRoID0gLjMNCmRhdGFTY2FubmVyc0VuaGFuY2VkICAlPiUgZ2dwbG90KGFlcyh4PXJlc3VsdCwgeT1leHRyYWN0b3IsIGZpbGw9ZXh0cmFjdG9yKSkgKyBzdGF0X3N1bW1hcnkoZnVuLmRhdGE9ZiwgZ2VvbT0iYm94cGxvdCIsd2lkdGg9Ym94d2lkdGgpICsgc3RhdF9ib3hwbG90KGdlb209J2Vycm9yYmFyJyxjb2VmPTEwLHdpZHRoPWJveHdpZHRoKSArIHN0YXRfc3VtbWFyeShmdW4uZGF0YSA9IG1lZGlhbl9jbF9ib290LCBnZW9tID0gImVycm9yYmFyIiwgY29sb3VyID0gImJsYWNrIiwgbGluZXR5cGU9IjIxIiwgd2lkdGg9LjcpICsgZ2VvbV9wb2ludChwY2g9MjEscG9zaXRpb24gPSBwb3NpdGlvbl9qaXR0ZXIod2lkdGg9MCwgaGVpZ2h0ID0gLjQpLGFscGhhPWppdHRlcnBvaW50YWxwaGEpICsgbGFicyh4PWV4cHJlc3Npb24oUltzXSksIHk9IkV4dHJhY3RvciIpICsgY29vcmRfY2FydGVzaWFuKHhsaW0gPSBjKDAuMCwxLjApLCBjbGlwPSJvZmYiKSArIHRoZW1lKGFzcGVjdC5yYXRpbz1hc3BlY3RyLCBsZWdlbmQucG9zaXRpb24gPSAibm9uZSIpDQpnZ3NhdmUoInNjYW5uZXJzLWJveHBsb3QucGRmIiwgZGV2aWNlPWNhaXJvX3BkZiwgd2lkdGg9cGFnZXdpZHRoLCBoZWlnaHQ9YXNwZWN0cipwYWdld2lkdGgsIHVuaXRzPXBhZ2V3aWR0aHVuaXQsIHNjYWxlPTEuMywgZHBpPTYwMCkNCmRhdGFDb25zZWN1dGl2ZUVuaGFuY2VkICU+JSBnZ3Bsb3QoYWVzKHg9cmVzdWx0LCB5PWV4dHJhY3RvciwgZmlsbD1leHRyYWN0b3IpKSArIHN0YXRfc3VtbWFyeShmdW4uZGF0YT1mLCBnZW9tPSJib3hwbG90Iix3aWR0aD1ib3h3aWR0aCkgKyBzdGF0X2JveHBsb3QoZ2VvbT0nZXJyb3JiYXInLGNvZWY9MTAsd2lkdGg9Ym94d2lkdGgpICsgc3RhdF9zdW1tYXJ5KGZ1bi5kYXRhID0gbWVkaWFuX2NsX2Jvb3QsIGdlb20gPSAiZXJyb3JiYXIiLCBjb2xvdXIgPSAiYmxhY2siLCBsaW5ldHlwZT0iMjEiLCB3aWR0aD0uNykgKyBnZW9tX3BvaW50KHBjaD0yMSxwb3NpdGlvbiA9IHBvc2l0aW9uX2ppdHRlcih3aWR0aD0wLCBoZWlnaHQgPSAuNCksYWxwaGE9aml0dGVycG9pbnRhbHBoYSkgKyBsYWJzKHg9ZXhwcmVzc2lvbihSW3NdKSwgeT0iRXh0cmFjdG9yIikgKyBjb29yZF9jYXJ0ZXNpYW4oeGxpbSA9IGMoMC42NSwxLjApLCBjbGlwPSJvZmYiKSArIHRoZW1lKGFzcGVjdC5yYXRpbz0wLjUsIGxlZ2VuZC5wb3NpdGlvbiA9ICJub25lIikNCmdnc2F2ZSgiY29uc2VjdXRpdmUtYm94cGxvdC5wZGYiLCBkZXZpY2U9Y2Fpcm9fcGRmLCB3aWR0aD1wYWdld2lkdGgsIGhlaWdodD1hc3BlY3RyKnBhZ2V3aWR0aCwgdW5pdHM9cGFnZXdpZHRodW5pdCwgc2NhbGU9MS4zLCBkcGk9NjAwKQ0KYGBgDQoNCkZvciBzY2FubmVycyB3ZSBhcmUgZXZlbnR1YWxseSBwcmVzZW50aW5nIG51bWJlciBvZiBtaXNzZWQgbWF0Y2hlcyBkaXJlY3RseSAoRmlndXJlIDMgLSBzY2FubmVycy1iYXJwbG90LnBkZikuDQoNCmBgYHtyfQ0KYXNwZWN0ciA9IC44DQpkYXRhU2Nhbm5lcnNFbmhhbmNlZCAlPiUgZ2dwbG90KGFlcyh4PXJlc3VsdCkpICsgZ2VvbV9iYXIod2lkdGg9LjAyLGFlcyhmaWxsPWV4dHJhY3RvcikpICsgZ2VvbV90ZXh0KHN0YXQ9J2NvdW50JywgYWVzKHk9Li5jb3VudC4uLGxhYmVsPS4uY291bnQuLiksIHZqdXN0PS0uNSwgc2l6ZT0yLjUpICsgZ2VvbV90ZXh0KGFlcyh5PS0zLGxhYmVsPW1pc3NlcyksIHZqdXN0PTEsIHNpemU9My4wKSArIGNvb3JkX2NhcnRlc2lhbih4bGltID0gYygwLjYsMS4wNSksIGNsaXA9Im9mZiIpICsgc2NhbGVfeF9jb250aW51b3VzKGJyZWFrcyA9IHNlcSgwLjEsIGJ5PTAuMSkpICsgc2NhbGVfeV9jb250aW51b3VzKGV4cGFuZCA9IGV4cGFuc2lvbihtdWx0ID0gYyguMSwgLjIpKSkgKyBndWlkZXMoZmlsbCA9IGd1aWRlX2xlZ2VuZChyZXZlcnNlID0gVFJVRSkpICsgZmFjZXRfd3JhcCh+ZmN0X3JldihleHRyYWN0b3IpLCBuY29sPTMsIGRyb3A9RkFMU0UpICsgbGFicyh4PWV4cHJlc3Npb24oUltzXSksIHk9IlJlc3VsdCBjb3VudCIpICsgdGhlbWUoYXNwZWN0LnJhdGlvPWFzcGVjdHIpDQpnZ3NhdmUoInNjYW5uZXJzLWJhcnBsb3QucGRmIiwgZGV2aWNlPWNhaXJvX3BkZiwgIHdpZHRoPXBhZ2V3aWR0aCwgaGVpZ2h0PS43KmFzcGVjdHIqcGFnZXdpZHRoLCB1bml0cz1wYWdld2lkdGh1bml0LCBzY2FsZT0xLjMsIGRwaT02MDApDQpgYGANCg0KVmlzdWFsaXphdGlvbiBvZiBkaXN0YW5jZSBtZWFzdXJlcyAtIGEgYm94cGxvdCBmb3IgZnVsbCBXU0lzIChGaWd1cmUgNSAtIGRpc3RhbmNlcy1ib3hwbG90LnBkZikgYW5kIGEgYm94cGxvdCBmb3IgY3JvcHBlZCBXU0lzIChGaWd1cmUgMTAgLSBkaXN0YW5jZXMtY3JvcC1ib3hwbG90LnBkZikuIE5vdGUgdGhhdCB2aW9saW4gcGxvdHMgYXJlIG5vdCB1c2VkIGluIHRoZSBwYXBlciBhcyB0aGV5IGFyZSBsZXNzIGNvbW1vbiB0eXBlIG9mIHZpc3VhbGl6YXRpb24gYW5kIHJlcXVpcmUgaGlnaGVyIHJlc29sdXRpb24gdG8gYmUgdmlld2VkIHByb3Blcmx5Lg0KDQpgYGB7cn0NCmJveHdpZHRoID0gLjUNCg0KYXNwZWN0ciA9IDAuNw0KZG9kZ2UgPSAwLjg1DQpkYXRhRGlzdGFuY2VzICU+JSBtdXRhdGUoZ3JvdXAgPSBwYXN0ZShkaXN0YW5jZSxleHRyYWN0b3Isc2VwPSctJykpICU+JSBncm91cF9ieShkaXN0YW5jZSkgJT4lIGdncGxvdChhZXMoeD1mYWN0b3IoZGlzdGFuY2UpLCB5PXJlc3VsdCxmaWxsPWV4dHJhY3RvcikpICsgc3RhdF9zdW1tYXJ5KGZ1bi5kYXRhPWYsIGdlb209ImJveHBsb3QiLHBvc2l0aW9uID0gcG9zaXRpb25fZG9kZ2Uod2lkdGg9ZG9kZ2UpLHdpZHRoPWJveHdpZHRoKSArIHN0YXRfYm94cGxvdChnZW9tPSdlcnJvcmJhcicsY29lZj0xMCxwb3NpdGlvbiA9IHBvc2l0aW9uX2RvZGdlKHdpZHRoPWRvZGdlKSx3aWR0aD1ib3h3aWR0aCkgKyBzdGF0X3N1bW1hcnkoZnVuLmRhdGEgPSBtZWRpYW5fY2xfYm9vdCwgZ2VvbSA9ICJlcnJvcmJhciIsIGNvbG91ciA9ICJibGFjayIsIGxpbmV0eXBlPSIyMSIscG9zaXRpb24gPSBwb3NpdGlvbl9kb2RnZSh3aWR0aD1kb2RnZSksIHdpZHRoPTEuMikgKyBnZW9tX3BvaW50KHBjaCA9IDIxLCBwb3NpdGlvbiA9IHBvc2l0aW9uX2ppdHRlcmRvZGdlKGppdHRlci53aWR0aCA9IC4xLCBkb2RnZS53aWR0aCA9IGRvZGdlKSwgYWxwaGE9aml0dGVycG9pbnRhbHBoYSkgKyBjb29yZF9mbGlwKCkgKyBzY2FsZV94X2Rpc2NyZXRlKGxhYmVscz1jKCIzIiA9ICIzIG1tICg3LjAgcC9wKSAiLCAiNiIgPSAiNiBtbSAoNS4zIHAvcCkiLCAiOSIgPSAiOSBtbSAoNC4wIHAvcCkiLCAiMTIiID0gIjEyIG1tICgyLjcgcC9wKSIsICIxNSIgPSAiMTUgbW0gKDIuMSBwL3ApIiwgIjE4IiA9ICIxOCBtbSAoMS40IHAvcCkiKSkgKyBsYWJzKHk9ZXhwcmVzc2lvbihSW3NdKSwgeD1leHByZXNzaW9uKCJNaW4uIGRpc3RhbmNlIHRocmVzaG9sZCAifml0YWxpYyhsKX4iKGF2ZyBudW1iZXIgb2YgcHJvYmVzIHBlciBwYXRpZW50KSIpKSArIGd1aWRlcyhmaWxsID0gZ3VpZGVfbGVnZW5kKHJldmVyc2UgPSBUUlVFLCBkaXJlY3Rpb24gPSAidmVydGljYWwiKSkgKyB0aGVtZShhc3BlY3QucmF0aW89YXNwZWN0cikNCmdnc2F2ZSgiZGlzdGFuY2VzLWJveHBsb3QucGRmIiwgZGV2aWNlPWNhaXJvX3BkZiwgd2lkdGg9cGFnZXdpZHRoLCBoZWlnaHQ9YXNwZWN0cipwYWdld2lkdGgsIHVuaXRzPXBhZ2V3aWR0aHVuaXQsIHNjYWxlPTEuNSwgZHBpPTYwMCkNCg0KYXNwZWN0ciA9IDMuMA0KZG9kZ2UgPSAwLjkNCmRhdGFEaXN0YW5jZXMgJT4lIG11dGF0ZShncm91cCA9IHBhc3RlKGRpc3RhbmNlLGV4dHJhY3RvcixzZXA9Jy0nKSkgJT4lIGdyb3VwX2J5KGRpc3RhbmNlKSAlPiUgZ2dwbG90KGFlcyh4PWZhY3RvcihkaXN0YW5jZSksIHk9cmVzdWx0LGZpbGw9ZXh0cmFjdG9yKSkgKyBnZW9tX3Zpb2xpbihwb3NpdGlvbiA9IHBvc2l0aW9uX2RvZGdlKHdpZHRoID0gZG9kZ2UpKSArIGdlb21fYm94cGxvdChvdXRsaWVyLnNoYXBlID0gTkEscG9zaXRpb24gPSBwb3NpdGlvbl9kb2RnZSh3aWR0aCA9IGRvZGdlKSxhbHBoYT0uNCkgKyBzdGF0X3N1bW1hcnkoZnVuLmRhdGEgPSBtZWRpYW5fY2xfYm9vdCwgZ2VvbSA9ICJlcnJvcmJhciIsIGNvbG91ciA9ICJibGFjayIsIGxpbmV0eXBlPSIyMSIscG9zaXRpb24gPSBwb3NpdGlvbl9kb2RnZSh3aWR0aD1kb2RnZSksIHdpZHRoPTEuMikgICsgZ2VvbV9wb2ludChwY2ggPSAyMSwgcG9zaXRpb24gPSBwb3NpdGlvbl9qaXR0ZXJkb2RnZShqaXR0ZXIud2lkdGggPSAuMSwgZG9kZ2Uud2lkdGggPSBkb2RnZSksIGFscGhhPWppdHRlcnBvaW50YWxwaGEpICsgY29vcmRfZmxpcCgpICsgc2NhbGVfeF9kaXNjcmV0ZShsYWJlbHM9YygiMyIgPSAiMyBtbSAoNy4wIHAvcCkgIiwgIjYiID0gIjYgbW0gKDUuMyBwL3ApIiwgIjkiID0gIjkgbW0gKDQuMCBwL3ApIiwgIjEyIiA9ICIxMiBtbSAoMi43IHAvcCkiLCAiMTUiID0gIjE1IG1tICgyLjEgcC9wKSIsICIxOCIgPSAiMTggbW0gKDEuNCBwL3ApIikpICsgbGFicyh5PWV4cHJlc3Npb24oUltzXSksIHg9ZXhwcmVzc2lvbigiTWluLiBkaXN0YW5jZSB0aHJlc2hvbGQgIn5pdGFsaWMobCl+IihhdmcgbnVtYmVyIG9mIHByb2JlcyBwZXIgcGF0aWVudCkiKSkgKyBndWlkZXMoZmlsbCA9IGd1aWRlX2xlZ2VuZChyZXZlcnNlID0gVFJVRSkpICsgdGhlbWUoYXNwZWN0LnJhdGlvPWFzcGVjdHIpDQpnZ3NhdmUoImRpc3RhbmNlcy12aW9saW5wbG90LnBkZiIsIGRldmljZT1jYWlyb19wZGYsIHdpZHRoPXBhZ2V3aWR0aCwgaGVpZ2h0PWFzcGVjdHIqcGFnZXdpZHRoLCB1bml0cz1wYWdld2lkdGh1bml0LCBzY2FsZT0yLjAsIGRwaT02MDApDQoNCmFzcGVjdHIgPSAwLjcNCmRvZGdlID0gMC44NQ0KZGF0YUNyb3BEaXN0YW5jZXMgJT4lIG11dGF0ZShncm91cCA9IHBhc3RlKGRpc3RhbmNlLGV4dHJhY3RvcixzZXA9Jy0nKSkgJT4lIGdyb3VwX2J5KGRpc3RhbmNlKSAlPiUgZ2dwbG90KGFlcyh4PWZhY3RvcihkaXN0YW5jZSksIHk9cmVzdWx0LGZpbGw9ZXh0cmFjdG9yKSkgKyBzdGF0X3N1bW1hcnkoZnVuLmRhdGE9ZiwgZ2VvbT0iYm94cGxvdCIscG9zaXRpb24gPSBwb3NpdGlvbl9kb2RnZSh3aWR0aD1kb2RnZSksd2lkdGg9Ym94d2lkdGgpICsgc3RhdF9ib3hwbG90KGdlb209J2Vycm9yYmFyJyxjb2VmPTEwLHBvc2l0aW9uID0gcG9zaXRpb25fZG9kZ2Uod2lkdGg9ZG9kZ2UpLHdpZHRoPWJveHdpZHRoKSArIHN0YXRfc3VtbWFyeShmdW4uZGF0YSA9IG1lZGlhbl9jbF9ib290LCBnZW9tID0gImVycm9yYmFyIiwgY29sb3VyID0gImJsYWNrIiwgbGluZXR5cGU9IjIxIixwb3NpdGlvbiA9IHBvc2l0aW9uX2RvZGdlKHdpZHRoPWRvZGdlKSwgd2lkdGg9MS4yKSArIGdlb21fcG9pbnQocGNoID0gMjEsIHBvc2l0aW9uID0gcG9zaXRpb25faml0dGVyZG9kZ2Uoaml0dGVyLndpZHRoID0gLjEsIGRvZGdlLndpZHRoID0gZG9kZ2UpLCBhbHBoYT1qaXR0ZXJwb2ludGFscGhhKSArIGNvb3JkX2ZsaXAoKSArIHNjYWxlX3hfZGlzY3JldGUobGFiZWxzPWMoIjMiID0gIjMgbW0gKDcuMCBwL3ApICIsICI2IiA9ICI2IG1tICg1LjMgcC9wKSIsICI5IiA9ICI5IG1tICg0LjAgcC9wKSIsICIxMiIgPSAiMTIgbW0gKDIuNyBwL3ApIiwgIjE1IiA9ICIxNSBtbSAoMi4xIHAvcCkiLCAiMTgiID0gIjE4IG1tICgxLjQgcC9wKSIpKSArIGxhYnMoeT1leHByZXNzaW9uKFJbc10pLCB4PWV4cHJlc3Npb24oIk1pbi4gZGlzdGFuY2UgdGhyZXNob2xkICJ+aXRhbGljKGwpfiIoYXZnIG51bWJlciBvZiBwcm9iZXMgcGVyIHBhdGllbnQpIikpICsgZ3VpZGVzKGZpbGwgPSBndWlkZV9sZWdlbmQocmV2ZXJzZSA9IFRSVUUsIGRpcmVjdGlvbiA9ICJ2ZXJ0aWNhbCIpKSArIHRoZW1lKGFzcGVjdC5yYXRpbz1hc3BlY3RyKQ0KZ2dzYXZlKCJkaXN0YW5jZXMtY3JvcC1ib3hwbG90LnBkZiIsIGRldmljZT1jYWlyb19wZGYsIHdpZHRoPXBhZ2V3aWR0aCwgaGVpZ2h0PWFzcGVjdHIqcGFnZXdpZHRoLCB1bml0cz1wYWdld2lkdGh1bml0LCBzY2FsZT0xLjUsIGRwaT02MDApDQoNCg0KYXNwZWN0ciA9IDMuMA0KZG9kZ2UgPSAwLjkNCmRhdGFDcm9wRGlzdGFuY2VzICU+JSBtdXRhdGUoZ3JvdXAgPSBwYXN0ZShkaXN0YW5jZSxleHRyYWN0b3Isc2VwPSctJykpICU+JSBncm91cF9ieShkaXN0YW5jZSkgJT4lIGdncGxvdChhZXMoeD1mYWN0b3IoZGlzdGFuY2UpLCB5PXJlc3VsdCxmaWxsPWV4dHJhY3RvcikpICsgZ2VvbV92aW9saW4ocG9zaXRpb24gPSBwb3NpdGlvbl9kb2RnZSh3aWR0aCA9IGRvZGdlKSkgKyBnZW9tX2JveHBsb3Qob3V0bGllci5zaGFwZSA9IE5BLHBvc2l0aW9uID0gcG9zaXRpb25fZG9kZ2Uod2lkdGggPSBkb2RnZSksYWxwaGE9LjQpICsgc3RhdF9zdW1tYXJ5KGZ1bi5kYXRhID0gbWVkaWFuX2NsX2Jvb3QsIGdlb20gPSAiZXJyb3JiYXIiLCBjb2xvdXIgPSAiYmxhY2siLCBsaW5ldHlwZT0iMjEiLHBvc2l0aW9uID0gcG9zaXRpb25fZG9kZ2Uod2lkdGg9ZG9kZ2UpLCB3aWR0aD0xLjIpICsgZ2VvbV9wb2ludChwY2ggPSAyMSwgcG9zaXRpb24gPSBwb3NpdGlvbl9qaXR0ZXJkb2RnZShqaXR0ZXIud2lkdGggPSAuMSwgZG9kZ2Uud2lkdGggPSBkb2RnZSksIGFscGhhPWppdHRlcnBvaW50YWxwaGEpICsgY29vcmRfZmxpcCgpICsgc2NhbGVfeF9kaXNjcmV0ZShsYWJlbHM9YygiMyIgPSAiMyBtbSAoNy4wIHAvcCkgIiwgIjYiID0gIjYgbW0gKDUuMyBwL3ApIiwgIjkiID0gIjkgbW0gKDQuMCBwL3ApIiwgIjEyIiA9ICIxMiBtbSAoMi43IHAvcCkiLCAiMTUiID0gIjE1IG1tICgyLjEgcC9wKSIsICIxOCIgPSAiMTggbW0gKDEuNCBwL3ApIikpICsgbGFicyh5PWV4cHJlc3Npb24oUltzXSksIHg9ZXhwcmVzc2lvbigiTWluLiBkaXN0YW5jZSB0aHJlc2hvbGQgIn5pdGFsaWMobCl+IihhdmcgbnVtYmVyIG9mIHByb2JlcyBwZXIgcGF0aWVudCkiKSkgKyBndWlkZXMoZmlsbCA9IGd1aWRlX2xlZ2VuZChyZXZlcnNlID0gVFJVRSkpICsgdGhlbWUoYXNwZWN0LnJhdGlvPWFzcGVjdHIpDQpnZ3NhdmUoImRpc3RhbmNlcy1jcm9wLXZpb2xpbnBsb3QucGRmIiwgZGV2aWNlPWNhaXJvX3BkZiwgd2lkdGg9cGFnZXdpZHRoLCBoZWlnaHQ9YXNwZWN0cipwYWdld2lkdGgsIHVuaXRzPXBhZ2V3aWR0aHVuaXQsIHNjYWxlPTIuMCwgZHBpPTYwMCkNCmBgYA0KQ2FsY3VsYXRpbmcgc3VjY2VzcyByYXRlIHN0YXRzIGZvciBub24tY29uc2VjdXRpdmUgc2xpZGVzICg9IG1heCBpbiB0aGUgdGFibGUpLiBOb3RlIHRoYXQgdGhlc2UgdGFibGVzIGFyZSBub3QgZGlyZWN0bHkgdXNlZCBpbiB0aGUgcGFwZXIuDQoNCmBgYHtyfQ0KZGF0YU51bWJlclBhdGllbnRzICU+JSBmaWx0ZXIoZXh0cmFjdG9yPT0iU2ltQ0xSdjIiKSAlPiUgZ3JvdXBfYnkobnVtYmVyX29mX3BhdGllbnRzKSAlPiUgc3VtbWFyaXplX2F0KHZhcnMocmVzdWx0KSwgbGlzdChtaW49bWluLCBRMT1+cXVhbnRpbGUoLiwgcHJvYnMgPSAwLjI1KSwgbWVkaWFuPW1lZGlhbiwgUTM9fnF1YW50aWxlKC4sIHByb2JzID0gMC43NSksIG1heD1tYXgpKQ0KDQpkYXRhRGlzdGFuY2VzICU+JSBncm91cF9ieShkaXN0YW5jZSkgJT4lIHN1bW1hcml6ZV9hdCh2YXJzKHJlc3VsdCksIGxpc3QobWluPW1pbiwgUTE9fnF1YW50aWxlKC4sIHByb2JzID0gMC4yNSksIG1lZGlhbj1tZWRpYW4sIFEzPX5xdWFudGlsZSguLCBwcm9icyA9IDAuNzUpLCBtYXg9bWF4KSkNCmRhdGFEaXN0YW5jZXNFcHNpbG9ucyA8LSBkYXRhRGlzdGFuY2VzICU+JSBncm91cF9ieShkaXN0YW5jZSkgJT4lIHN1bW1hcml6ZV9hdCh2YXJzKHJlc3VsdCksIGxpc3QobWF4PW1heCkpICU+JSBtdXRhdGUoZGlzdGFuY2UgPSBmYWN0b3IoZGlzdGFuY2UpLCBtYXggPSBzcHJpbnRmKCIlMC4zZiIsIHJvdW5kLm9mZihtYXgsMykpKSAlPiUgcmVuYW1lX2F0KHZhcnMoYygiZGlzdGFuY2UiLCAibWF4IikpLCB+IGMoImRpc3RhbmNlIFttbV0iLCAnJFxcdmFyZXBzaWxvbiQnKSklPiUgdCgpDQpsYXRleFRhYmxlIDwtIHh0YWJsZShkYXRhRGlzdGFuY2VzRXBzaWxvbnMsIGNhcHRpb24gPSAiVmFsdWVzIG9mICRcXHZhcmVwc2lsb24kIGZvciBub24tY29uc2VjdXRpdmUgc2xpZGVzLiIsIGxhYmVsPSJ0YWI6cmVzdWx0czpub24tY29uc2VjdXRpdmU6ZXBzaWxvbiIpDQojYWxpZ24obGF0ZXhUYWJsZSkgJTw+JSBzdHJfcmVwbGFjZSgibCIsICJTW3RhYmxlLWZvcm1hdD0wLjNdfCIpDQojYWxpZ24obGF0ZXhUYWJsZSkgPC0geGFsaWduKGxhdGV4VGFibGUpDQojZGlnaXRzKGxhdGV4VGFibGUpIDwtIHhkaWdpdHMobGF0ZXhUYWJsZSkNCiNkaXNwbGF5KGxhdGV4VGFibGUpIDwtIHhkaXNwbGF5KGxhdGV4VGFibGUpDQpwcmludChsYXRleFRhYmxlLGZpbGU9InRhYi1yZXN1bHRzLW5vbmNvbnNlY3V0aXZlLWVwc2lsb24udGV4IixhcHBlbmQ9VCx0YWJsZS5wbGFjZW1lbnQgPSAiaGJ0cCIsY2FwdGlvbi5wbGFjZW1lbnQ9ImJvdHRvbSIsIGhsaW5lLmFmdGVyPXNlcShmcm9tPTAsdG89bnJvdyhsYXRleFRhYmxlKSxieT0xKSwgaW5jbHVkZS5jb2xuYW1lcyA9IEZBTFNFLCBzYW5pdGl6ZS50ZXh0LmZ1bmN0aW9uID0gZnVuY3Rpb24oeCl7eH0pDQoNCmRhdGFDcm9wRGlzdGFuY2VzICU+JSBncm91cF9ieShkaXN0YW5jZSkgJT4lIHN1bW1hcml6ZV9hdCh2YXJzKHJlc3VsdCksIGxpc3QobWluPW1pbiwgUTE9fnF1YW50aWxlKC4sIHByb2JzID0gMC4yNSksIG1lZGlhbj1tZWRpYW4sIFEzPX5xdWFudGlsZSguLCBwcm9icyA9IDAuNzUpLCBtYXg9bWF4KSkNCmRhdGFDcm9wRGlzdGFuY2VzRXBzaWxvbnMgPC0gZGF0YUNyb3BEaXN0YW5jZXMgJT4lIGdyb3VwX2J5KGRpc3RhbmNlKSAlPiUgc3VtbWFyaXplX2F0KHZhcnMocmVzdWx0KSwgbGlzdChtYXg9bWF4KSkgJT4lIG11dGF0ZShkaXN0YW5jZSA9IGZhY3RvcihkaXN0YW5jZSksIG1heCA9IHNwcmludGYoIiUwLjNmIiwgcm91bmQub2ZmKG1heCwzKSkpICU+JSByZW5hbWVfYXQodmFycyhjKCJkaXN0YW5jZSIsICJtYXgiKSksIH4gYygiZGlzdGFuY2UgW21tXSIsICckXFx2YXJlcHNpbG9uJCcpKSU+JSB0KCkNCmxhdGV4VGFibGUgPC0geHRhYmxlKGRhdGFDcm9wRGlzdGFuY2VzRXBzaWxvbnMsIGNhcHRpb24gPSAiVmFsdWVzIG9mICRcXHZhcmVwc2lsb24kIGZvciBub24tY29uc2VjdXRpdmUgY3JvcHBlZCBzbGlkZXMuIiwgbGFiZWw9InRhYjpyZXN1bHRzOm5vbi1jb25zZWN1dGl2ZTplcHNpbG9uIikNCiNhbGlnbihsYXRleFRhYmxlKSAlPD4lIHN0cl9yZXBsYWNlKCJsIiwgIlNbdGFibGUtZm9ybWF0PTAuM118IikNCiNhbGlnbihsYXRleFRhYmxlKSA8LSB4YWxpZ24obGF0ZXhUYWJsZSkNCiNkaWdpdHMobGF0ZXhUYWJsZSkgPC0geGRpZ2l0cyhsYXRleFRhYmxlKQ0KI2Rpc3BsYXkobGF0ZXhUYWJsZSkgPC0geGRpc3BsYXkobGF0ZXhUYWJsZSkNCnByaW50KGxhdGV4VGFibGUsZmlsZT0idGFiLXJlc3VsdHMtbm9uY29uc2VjdXRpdmUtY3JvcHMtZXBzaWxvbi50ZXgiLGFwcGVuZD1ULHRhYmxlLnBsYWNlbWVudCA9ICJoYnRwIixjYXB0aW9uLnBsYWNlbWVudD0iYm90dG9tIiwgaGxpbmUuYWZ0ZXI9c2VxKGZyb209MCx0bz1ucm93KGxhdGV4VGFibGUpLGJ5PTEpLCBpbmNsdWRlLmNvbG5hbWVzID0gRkFMU0UsIHNhbml0aXplLnRleHQuZnVuY3Rpb24gPSBmdW5jdGlvbih4KXt4fSkNCmBgYA0KDQoNClZpc3VhbGl6YXRpb24gb2YgY3JvcCBzaGlmdHMgKEZpZ3VyZSA5IC0gY3JvcHNoaWZ0cy1ib3hwbG90LnBkZikuIE5vdGUgdGhhdCB2aW9saW4gcGxvdCBpcyBub3QgdXNlZCBpbiB0aGUgcGFwZXIgYXMgdGhleSBhcmUgbGVzcyBjb21tb24gdHlwZSBvZiB2aXN1YWxpemF0aW9uLg0KDQpgYGB7cn0NCmFzcGVjdHIgPSAwLjcNCmRvZGdlID0gMC43NQ0KYm94d2lkdGggPSAuNQ0KZGF0YUNyb3BTaGlmdHMgJT4lIGdyb3VwX2J5KHNoaWZ0LGV4dHJhY3RvcikgJT4lIHN1bW1hcml6ZV9hdCh2YXJzKHJlc3VsdCksIGxpc3QobWluPW1pbiwgUTE9fnF1YW50aWxlKC4sIHByb2JzID0gMC4yNSksIG1lZGlhbj1tZWRpYW4sIFEzPX5xdWFudGlsZSguLCBwcm9icyA9IDAuNzUpLCBtYXg9bWF4KSkNCiNkYXRhQ3JvcFNoaWZ0cyAlPiUgbXV0YXRlKGdyb3VwID0gcGFzdGUoc2hpZnQsZXh0cmFjdG9yLHNlcD0nLScpKSAlPiUgZ3JvdXBfYnkoc2hpZnQpICU+JSBnZ3Bsb3QoYWVzKHg9ZmFjdG9yKHNoaWZ0KSwgeT1yZXN1bHQsZmlsbD1leHRyYWN0b3IpKSArIGdlb21fYm94cGxvdChvdXRsaWVyLnNoYXBlID0gTkEscG9zaXRpb24gPSBwb3NpdGlvbl9kb2RnZTIoKSkgKyBnZW9tX3BvaW50KHBjaCA9IDIxLCBwb3NpdGlvbiA9IHBvc2l0aW9uX2ppdHRlcmRvZGdlKGppdHRlci53aWR0aCA9IC4xKSwgYWxwaGE9aml0dGVycG9pbnRhbHBoYSkgKyBjb29yZF9mbGlwKCkgKyBzY2FsZV94X2Rpc2NyZXRlKGxhYmVscz1jKCIwIiA9ICIwIHB4ICgxMDAlKSAiLCAiNSIgPSAiNSBweCAoOTctOTglKSIsICIxMCIgPSAiMTAgcHggKDk0LTk2JSkiLCAiMTUiID0gIjE1IHB4ICg5MS05MyUpIiwgIjI1IiA9ICIyNSBweCAoODUtODklKSIsICI1MCIgPSAiNTAgcHggKDcxLTc4JSkiLCAiNzUiID0gIjc1IHB4ICg1OC02NyUpIiwgIjEwMCIgPSAiMTAwIHB4ICg0Ny01NSUpIikpICsgbGFicyh5PWV4cHJlc3Npb24oUltzXSksIHg9IlNoaWZ0IChvdmVybGFwKSIpICsgZ3VpZGVzKGZpbGwgPSBndWlkZV9sZWdlbmQocmV2ZXJzZSA9IFRSVUUpKSArIHRoZW1lKGFzcGVjdC5yYXRpbz1hc3BlY3RyKQ0KZGF0YUNyb3BTaGlmdHMgJT4lIG11dGF0ZShncm91cCA9IHBhc3RlKHNoaWZ0LGV4dHJhY3RvcixzZXA9Jy0nKSkgJT4lIGdyb3VwX2J5KHNoaWZ0KSAlPiUgZ2dwbG90KGFlcyh4PWZhY3RvcihzaGlmdCksIHk9cmVzdWx0LGZpbGw9ZXh0cmFjdG9yKSkgKyBzdGF0X3N1bW1hcnkoZnVuLmRhdGE9ZiwgZ2VvbT0iYm94cGxvdCIscG9zaXRpb24gPSBwb3NpdGlvbl9kb2RnZSh3aWR0aD1kb2RnZSksd2lkdGg9Ym94d2lkdGgpICsgc3RhdF9ib3hwbG90KGdlb209J2Vycm9yYmFyJyxjb2VmPTEwLHBvc2l0aW9uID0gcG9zaXRpb25fZG9kZ2Uod2lkdGg9ZG9kZ2UpLHdpZHRoPWJveHdpZHRoKSAgKyBzdGF0X3N1bW1hcnkoZnVuLmRhdGEgPSBtZWRpYW5fY2xfYm9vdCwgZ2VvbSA9ICJlcnJvcmJhciIsIGNvbG91ciA9ICJibGFjayIsIGxpbmV0eXBlPSIyMSIscG9zaXRpb24gPSBwb3NpdGlvbl9kb2RnZSh3aWR0aD1kb2RnZSksIHdpZHRoPTEuMikgICsgZ2VvbV9wb2ludChwY2ggPSAyMSwgcG9zaXRpb24gPSBwb3NpdGlvbl9qaXR0ZXJkb2RnZShqaXR0ZXIud2lkdGggPSAuMSksIGFscGhhPWppdHRlcnBvaW50YWxwaGEqLjUpICsgY29vcmRfZmxpcCgpICsgc2NhbGVfeF9kaXNjcmV0ZShsYWJlbHM9YygiMCIgPSAiMCBweCAoMTAwJSkgIiwgIjUiID0gIjUgcHggKDk3LTk4JSkiLCAiMTAiID0gIjEwIHB4ICg5NC05NiUpIiwgIjE1IiA9ICIxNSBweCAoOTEtOTMlKSIsICIyNSIgPSAiMjUgcHggKDg1LTg5JSkiLCAiNTAiID0gIjUwIHB4ICg3MS03OCUpIiwgIjc1IiA9ICI3NSBweCAoNTgtNjclKSIsICIxMDAiID0gIjEwMCBweCAoNDctNTUlKSIpKSArIGxhYnMoeT1leHByZXNzaW9uKFJbc10pLCB4PSJTaGlmdCAob3ZlcmxhcCkiKSArIGd1aWRlcyhmaWxsID0gZ3VpZGVfbGVnZW5kKHJldmVyc2UgPSBUUlVFKSkgKyB0aGVtZShhc3BlY3QucmF0aW89YXNwZWN0cikNCmdnc2F2ZSgiY3JvcHNoaWZ0cy1ib3hwbG90LnBkZiIsIGRldmljZT1jYWlyb19wZGYsIHdpZHRoPXBhZ2V3aWR0aCwgaGVpZ2h0PWFzcGVjdHIqcGFnZXdpZHRoLCB1bml0cz1wYWdld2lkdGh1bml0LCBzY2FsZT0yLjAsIGRwaT02MDApDQoNCmFzcGVjdHIgPSAzLjANCmRvZGdlID0gMC45DQpkYXRhQ3JvcFNoaWZ0cyAlPiUgbXV0YXRlKGdyb3VwID0gcGFzdGUoc2hpZnQsZXh0cmFjdG9yLHNlcD0nLScpKSAlPiUgZ3JvdXBfYnkoc2hpZnQpICU+JSBnZ3Bsb3QoYWVzKHg9ZmFjdG9yKHNoaWZ0KSwgeT1yZXN1bHQsZmlsbD1leHRyYWN0b3IpKSArIGdlb21fdmlvbGluKHBvc2l0aW9uID0gcG9zaXRpb25fZG9kZ2Uod2lkdGggPSBkb2RnZSkpICArIGdlb21fcG9pbnQocGNoID0gMjEsIHBvc2l0aW9uID0gcG9zaXRpb25faml0dGVyZG9kZ2Uoaml0dGVyLndpZHRoID0gLjEpLCBhbHBoYT1qaXR0ZXJwb2ludGFscGhhKi41KSArIGNvb3JkX2ZsaXAoKSArIHNjYWxlX3hfZGlzY3JldGUobGFiZWxzPWMoIjAiID0gIjAgcHggKDEwMCUpICIsICI1IiA9ICI1IHB4ICg5Ny05OCUpIiwgIjEwIiA9ICIxMCBweCAoOTQtOTYlKSIsICIxNSIgPSAiMTUgcHggKDkxLTkzJSkiLCAiMjUiID0gIjI1IHB4ICg4NS04OSUpIiwgIjUwIiA9ICI1MCBweCAoNzEtNzglKSIsICI3NSIgPSAiNzUgcHggKDU4LTY3JSkiLCAiMTAwIiA9ICIxMDAgcHggKDQ3LTU1JSkiKSkgKyBsYWJzKHk9ZXhwcmVzc2lvbihSW3NdKSwgeD0iU2hpZnQgKG92ZXJsYXApIikgKyBndWlkZXMoZmlsbCA9IGd1aWRlX2xlZ2VuZChyZXZlcnNlID0gVFJVRSkpICsgdGhlbWUoYXNwZWN0LnJhdGlvPWFzcGVjdHIpDQpnZ3NhdmUoImNyb3BzaGlmdHMtdmlvbGlucGxvdC5wZGYiLCBkZXZpY2U9Y2Fpcm9fcGRmLCB3aWR0aD1wYWdld2lkdGgsIGhlaWdodD1hc3BlY3RyKnBhZ2V3aWR0aCwgdW5pdHM9cGFnZXdpZHRodW5pdCwgc2NhbGU9Mi4wLCBkcGk9NjAwKQ0KYGBgDQoNCk1ha2luZyBmYW5wbG90cyBvZiBudW1iZXJzIG9mIHBhdGllbnRzIGFuZCBzbGlkZXMgKEZpZ3VyZSA4YSAtIG51bWJlcnBhdGllbnRzLWludGVydmFscGxvdC5wZGYgYW5kIEZpZ3VyZSA4YiAtIG51bWJlcnNsaWRlcy1pbnRlcnZhbHBsb3QucGRmKS4gTm90ZSB0aGF0IHRoZSBib3hwbG90IGlzIG9ubHkgdXNlZCBmb3IgY29uc2lzdGVuY3kgY2hlY2sgYW5kIGlzIG5vdCB1c2VkIGluIHRoZSBwYXBlci4NCg0KYGBge3J9DQphc3BlY3RyID0gMC41DQpkb2RnZSA9IDAuNzUNCmJveHdpZHRoID0gLjUNCmxlZ2VuZHhzaGlmdCA9IC0uNQ0KbGVnZW5keXNoaWZ0ID0gLTIuMA0KZGF0YU51bWJlclNsaWRlcyAlPiUgZ3JvdXBfYnkoZXh0cmFjdG9yKSAlPiUgZ2dwbG90KGFlcyh4PWZhY3RvcihudW1iZXJfb2Zfc2xpZGVzKSwgeT1yZXN1bHQsIGZpbGw9ZXh0cmFjdG9yKSkgKyBzdGF0X3N1bW1hcnkoZnVuLmRhdGE9ZiwgZ2VvbT0iYm94cGxvdCIscG9zaXRpb24gPSBwb3NpdGlvbl9kb2RnZSh3aWR0aD1kb2RnZSksd2lkdGg9Ym94d2lkdGgpICsgc3RhdF9ib3hwbG90KGdlb209J2Vycm9yYmFyJyxjb2VmPTEwLHBvc2l0aW9uID0gcG9zaXRpb25fZG9kZ2Uod2lkdGg9ZG9kZ2UpLHdpZHRoPWJveHdpZHRoKSArIHN0YXRfc3VtbWFyeShmdW4uZGF0YSA9IG1lZGlhbl9jbF9ib290LCBnZW9tID0gImVycm9yYmFyIiwgY29sb3VyID0gImJsYWNrIiwgbGluZXR5cGU9IjIxIixwb3NpdGlvbiA9IHBvc2l0aW9uX2RvZGdlKHdpZHRoPWRvZGdlKSwgd2lkdGg9MS4yKSArIGdlb21fcG9pbnQocGNoID0gMjEsIHBvc2l0aW9uID0gcG9zaXRpb25faml0dGVyZG9kZ2Uoaml0dGVyLndpZHRoID0gLjEpLCBhbHBoYT1qaXR0ZXJwb2ludGFscGhhKSsgbGFicyh5PWV4cHJlc3Npb24oUltzXSksIHg9Ik51bWJlciBvZiBwcm9iZXMgcGVyIHBhdGllbnQiKSArIGd1aWRlcyhmaWxsID0gZ3VpZGVfbGVnZW5kKHJldmVyc2UgPSBUUlVFKSkgKyB0aGVtZShhc3BlY3QucmF0aW89YXNwZWN0cikNCmdnc2F2ZSgibnVtYmVyc2xpZGVzLWJveHBsb3QucGRmIiwgZGV2aWNlPWNhaXJvX3BkZiwgd2lkdGg9cGFnZXdpZHRoLCBoZWlnaHQ9YXNwZWN0cipwYWdld2lkdGgsIHVuaXRzPXBhZ2V3aWR0aHVuaXQsIHNjYWxlPTIuMCwgZHBpPTYwMCkNCmdnTnVtYmVyU2xpZGVzIDwtIGRhdGFOdW1iZXJTbGlkZXMgJT4lIGdyb3VwX2J5KGV4dHJhY3RvcikgJT4lIGdncGxvdChhZXMoeD1udW1iZXJfb2Zfc2xpZGVzLCB5PXJlc3VsdCwgZ3JvdXA9ZXh0cmFjdG9yKSkgKyBnZW9tX2ZhbihhZXMoZmlsbD1leHRyYWN0b3IsYWxwaGE9Li5JbnRlcnZhbC4uKSwgaW50ZXJ2YWxzPWMoLjUsMS4wKSwgYWxwaGE9LjMsc2hvdy5sZWdlbmQ9RkFMU0UpICsgZ2VvbV9pbnRlcnZhbChpbnRlcnZhbHM9YygwLC41LDEuMCkpICsgc2NhbGVfbGluZXR5cGVfbWFudWFsKHZhbHVlcz1jKCdzb2xpZCcsJ2RvdHRlZCcsJ2Rhc2hlZCcpLCBsYWJlbHM9YygnTWVkaWFuJywnUTEtUTMnLCAnbWluLW1heCcpKSArIGZhY2V0X3dyYXAofmZjdF9yZXYoZXh0cmFjdG9yKSwgbmNvbD0zLCBkcm9wPUZBTFNFKSArIGxhYnMoeT1leHByZXNzaW9uKFJbc10pLCB4PSJOdW1iZXIgb2YgcHJvYmVzIHBlciBwYXRpZW50IikgKyBndWlkZXMoZmlsbCA9IGd1aWRlX2xlZ2VuZChyZXZlcnNlID0gVFJVRSkpICsgdGhlbWUoYXNwZWN0LnJhdGlvPWFzcGVjdHIpICsgdGhlbWUobGVnZW5kLmRpcmVjdGlvbj0iaG9yaXpvbnRhbCIsIGxlZ2VuZC5wb3NpdGlvbj0iYm90dG9tIikNCiNnZ3Bsb3RHcm9iKGdnTnVtYmVyU2xpZGVzKQ0KI3NldF9sYXN0X3Bsb3QocmVwb3NpdGlvbl9sZWdlbmQoZ2dOdW1iZXJTbGlkZXMsICdib3R0b20gcmlnaHQnLCBwYW5lbCA9ICdwYW5lbC0zLTInKSkNCiNzZXRfbGFzdF9wbG90KHJlcG9zaXRpb25fbGVnZW5kKGdnTnVtYmVyUGF0aWVudHMsICdjZW50ZXInLCBwYW5lbCA9ICdwYW5lbC0yLTInLCB4PWxlZ2VuZHhzaGlmdCwgeT1sZWdlbmR5c2hpZnQpKQ0KZ2dOdW1iZXJTbGlkZXMNCmdnc2F2ZSgibnVtYmVyc2xpZGVzLWludGVydmFscGxvdC5wZGYiLCBkZXZpY2U9Y2Fpcm9fcGRmLCB3aWR0aD1wYWdld2lkdGgsIGhlaWdodD1hc3BlY3RyKnBhZ2V3aWR0aCwgdW5pdHM9cGFnZXdpZHRodW5pdCwgc2NhbGU9Mi4wLCBkcGk9NjAwKQ0KIyhnZ051bWJlclBhdGllbnRzIDwtIGRhdGFOdW1iZXJQYXRpZW50cyAlPiUgZ3JvdXBfYnkoZXh0cmFjdG9yKSAlPiUgZ2dwbG90KGFlcyh4PW51bWJlcl9vZl9wYXRpZW50cywgeT1yZXN1bHQsIGdyb3VwPWV4dHJhY3RvcikpICsgZ2VvbV9mYW4oYWVzKGZpbGw9ZXh0cmFjdG9yLGFscGhhPS4uSW50ZXJ2YWwuLiksIGludGVydmFscz1jKC41LDEuMCksIGFscGhhPS4zLHNob3cubGVnZW5kPUZBTFNFKSArIGdlb21faW50ZXJ2YWwoaW50ZXJ2YWxzPWMoMCwuNSwxLjApKSArIHNjYWxlX2xpbmV0eXBlX21hbnVhbCh2YWx1ZXM9Yygnc29saWQnLCdkb3R0ZWQnLCdkYXNoZWQnKSwgbGFiZWxzPWMoJ01lZGlhbicsJzI1LTc1JScsICdtaW4tbWF4JykpICsgZmFjZXRfd3JhcCh+ZmN0X3JldihleHRyYWN0b3IpLCBuY29sPTMsIGRyb3A9RkFMU0UpICsgbGFicyh5PWV4cHJlc3Npb24oUltzXSksIHg9Ik51bWJlciBvZiBwYXRpZW50cyIpICsgZ3VpZGVzKGZpbGwgPSBndWlkZV9sZWdlbmQocmV2ZXJzZSA9IFRSVUUpKSArIHRoZW1lKGFzcGVjdC5yYXRpbz1hc3BlY3RyKSkNCmdnTnVtYmVyUGF0aWVudHMgPC0gZGF0YU51bWJlclBhdGllbnRzICU+JSBncm91cF9ieShleHRyYWN0b3IpICU+JSBnZ3Bsb3QoYWVzKHg9bnVtYmVyX29mX3BhdGllbnRzLCB5PXJlc3VsdCwgZ3JvdXA9ZXh0cmFjdG9yKSkgKyBnZW9tX2ZhbihhZXMoZmlsbD1leHRyYWN0b3IsYWxwaGE9Li5JbnRlcnZhbC4uKSwgaW50ZXJ2YWxzPWMoLjUsMS4wKSwgYWxwaGE9LjMsc2hvdy5sZWdlbmQ9RkFMU0UpICsgZ2VvbV9pbnRlcnZhbChpbnRlcnZhbHM9YygwLC41LDEuMCkpICsgc2NhbGVfbGluZXR5cGVfbWFudWFsKHZhbHVlcz1jKCdzb2xpZCcsJ2RvdHRlZCcsJ2Rhc2hlZCcpLCBsYWJlbHM9YygnTWVkaWFuJywnUTEtUTMnLCAnbWluLW1heCcpKSArIGZhY2V0X3dyYXAofmZjdF9yZXYoZXh0cmFjdG9yKSwgbmNvbD0zLCBkcm9wPUZBTFNFKSArIGxhYnMoeT1leHByZXNzaW9uKFJbc10pLCB4PSJOdW1iZXIgb2YgcGF0aWVudHMiKSArIGd1aWRlcyhmaWxsID0gZ3VpZGVfbGVnZW5kKHJldmVyc2UgPSBUUlVFKSkgKyB0aGVtZShhc3BlY3QucmF0aW89YXNwZWN0cikgKyB0aGVtZShsZWdlbmQuZGlyZWN0aW9uPSJob3Jpem9udGFsIiwgbGVnZW5kLnBvc2l0aW9uPSJib3R0b20iKQ0KI2dncGxvdEdyb2IoZ2dOdW1iZXJQYXRpZW50cykNCiNzZXRfbGFzdF9wbG90KHJlcG9zaXRpb25fbGVnZW5kKGdnTnVtYmVyUGF0aWVudHMsICdib3R0b20gcmlnaHQnLCBwYW5lbCA9ICdwYW5lbC0zLTInLCB5PS0xLjIpKQ0KI3NldF9sYXN0X3Bsb3QocmVwb3NpdGlvbl9sZWdlbmQoZ2dOdW1iZXJQYXRpZW50cywgJ2NlbnRlcicsIHBhbmVsID0gJ3BhbmVsLTItMicsIHg9bGVnZW5keHNoaWZ0LCB5PWxlZ2VuZHlzaGlmdCkpDQpnZ051bWJlclBhdGllbnRzDQpnZ3NhdmUoIm51bWJlcnBhdGllbnRzLWludGVydmFscGxvdC5wZGYiLCBkZXZpY2U9Y2Fpcm9fcGRmLCB3aWR0aD1wYWdld2lkdGgsIGhlaWdodD1hc3BlY3RyKnBhZ2V3aWR0aCwgdW5pdHM9cGFnZXdpZHRodW5pdCwgc2NhbGU9Mi4wLCBkcGk9NjAwKQ0KYGBgDQo=
